# Changes in Alcohol Use during the COVID-19 Pandemic among Young Adults: The Prospective Effect of Anxiety and Depression

**DOI:** 10.3390/jcm10194468

**Published:** 2021-09-28

**Authors:** Belén del Valle Vera, José Carmona-Márquez, Óscar Martín Lozano-Rojas, Alberto Parrado-González, Claudio Vidal-Giné, Ricardo Marcos Pautassi, Fermín Fernández-Calderón

**Affiliations:** 1 Faculty of Psychology, National University of Córdoba, Córdoba X5000HUA, Argentina; belen.vera@unc.edu.ar (B.d.V.V.); ricardo.pautassi@unc.edu.ar (R.M.P.); 2 Institute of Psychological Research, IIPsi-CONICET-UNC, Córdoba X5000HUA, Argentina; 3 Department of Clinical and Experimental Psychology, University of Huelva, 21071 Huelva, Spain; carmona@uhu.es (J.C.-M.); oscar.lozano@dpsi.uhu.es (Ó.M.L.-R.); alberto.parrado@dpces.uhu.es (A.P.-G.); 4 Research Center on Natural Resources, Health and the Environment, University of Huelva, 21071 Huelva, Spain; 5 Non-Governmental Organization Welfare and Development, Energy Control, 29200 Antequera, Spain; claudiovidal@energycontrol.org; 6 Instituto de Investigación Médica M. y M. Ferreyra (INIMEC–CONICET-Universidad Nacional de Córdoba), Córdoba X5016NST, Argentina

**Keywords:** COVID-19, longitudinal, alcohol use, mental health status, young adults

## Abstract

Health measures instantiated to reduce the spread of COVID-19 have imposed significant constraints for the population and impacted on drinking habits and mental health. This study longitudinally compared changes in alcohol consumption before and after the COVID-19 outbreak and the impact of sociodemographic and mental health variables on such changes among a community sample of young adults. Data were collected in the context of a larger, ongoing longitudinal study. The sample consisted of 305 young adults from Spain aged between 18 and 26 years (mean age = 21.27, (*SD* = 2.21), female = 53.4%; college students = 61.6%) who completed first (November-2019 and February-2020; i.e., before the outbreak of COVID-19) and second follow-up questionnaires (March 2021, a year after the COVID-19 outbreak). Alcohol use (quantity and drinking frequency), depression and anxiety symptoms were measured. Quantity and frequency of alcohol use decreased from the pre- to post-COVID-19 period. A decrease in drinking frequency was observed among college students, but not in noncollege peers. Although we found no effect of pre-COVID-19 anxiety on alcohol use changes, those with more depressive symptoms at the pre-COVID assessment were more resistant to decreasing their drinking quantity and frequency after the COVID-19 outbreak. This information will be of value when designing interventions aimed at reducing harmful alcohol use and highlights the role of mental health status when identifying high risk populations of young-adults during this, and future, public health crises.

## 1. Introduction

Alcohol use is a relevant public health concern around the world [1] and has been pointed out as one of the leading causes of preventable disease, death and disability [2]. Excessive alcohol consumption is more prevalent among young people than in any other age group [3,4,5,6]. Compared to adults, young people drink more per drinking occasion, although less frequently [7,8,9], and exhibit a greater frequency of hazardous drinking, including binge drinking [2,10], which has been associated with immediate, e.g., academic problems, risky sexual behaviors, physical and sexual assaults, violent behavior and traffic accidents [11,12,13], and long-term negative consequences, including increased probability of developing alcohol use disorders [14,15]. In Spain, it is estimated that 79.4% and 61.8% of people aged 15–24 have consumed alcohol during the last year and last month, respectively. In comparison to other age groups, people aged 15–24 showed the highest prevalence of alcohol intoxication (i.e., drunkenness) during the last year (38.3%) and last month (15.7%) [4]. Drinking patterns differ according to sex, with men reporting higher quantity and frequency of alcohol use, and more frequent binge drinking than women [2], although there are reports indicating this gap may have been (at least in the US) decreasing in the last decade [16].

COVID-19 is an infectious disease caused by a newly discovered coronavirus that reached pandemic levels in March 2020 and has been declared a Public Health Emergency of International Concern by the World Health Organization [17]. In Spain, regional governments implemented a variety of sanitary measures such as mandatory home quarantine, social distancing measures, closure of nonessential premises, restricted mobility and closures of the educational system (including college) to slow down the spread of the virus. These measures have imposed significant environmental, contextual, and social constraints on the population, resulting in changes to daily life activities and social interactions, which seems to have affected drinking habits and mental health [18,19,20,21,22].

Studies examining changes in alcohol use during the pandemic among young adults have reported mixed results. Some studies reported an increase in quantity and frequency of alcohol use [23,24], but others found a decrease in these indicators [25,26,27,28]. Moreover, mixed results have been reported within the same sample when different alcohol outcomes were considered [29,30]. For example, in a study carried out with US college students [30], a decrease was found both in the typical number of drinks per week and in maximum drinks per day, although a slight increase in drinking frequency was observed. Finally, some studies have shown a greater decrease [28,31,32] in alcohol use among young people than in other age groups. Mixed results have also been reported in studies conducted among the general adult population [33,34,35]. In general, the studies that reported increases in alcohol use during the pandemic argued that this may have been due to increased stress, loss of daily structure, greater time availability, or boredom [36,37,38,39]. On the other hand, diminished alcohol availability and income reduction due to the economic crisis derived from the pandemic, have been some of the factors associated with alcohol use reductions [36].

These mixed results may be a consequence of the different methodologies employed to assess alcohol consumption and to compare alcohol use before and after the COVID-19 outbreak. It should be noted that, mainly due to the urgency of the matter and the impossibility of planning pre and post longitudinal studies to assess the impact of the pandemic on different behaviors, most studies relied on retrospective self-reported data on alcohol use before and during the pandemic [24,25,28,31,33,35]. Moreover, some studies [29,32] simply asked about self-perceived changes in alcohol consumption (i.e., whether participants drank more, the same or less than before COVID-19) without including prior objective measures of drinking quantity and frequency. For instance, in one such study, participants (Uruguayans 18 to 60 years old) were asked if they believed they had increased the volume of use of their preferred substance after the instantiation of the COVID-19 related quarantine [40]. These approaches may be susceptible to recall bias, affecting the validity of their results. It has been pointed out that the reliability of recall decreases as the complexity of the recall task increases (e.g., broad reference periods [41,42]). Moreover, these measurements are also affected by telescopic biases, by which recent behaviors are perceived as more remote than they actually are, whereas distant events are reported to have occurred closer in time [43].

To our knowledge, only five studies have longitudinally analyzed how alcohol use changed, using measurement milestones timepoints located before and after de pandemic [26,27,30,34,44]. Three of these [26,27,30] were carried out with samples of college students from the US or UK. Given that alcohol consumption is central to college culture [13,45,46,47], and that college life in those countries exhibit idiosyncratic characteristics, e.g., presence of social organizations that actively promote alcohol use (see [48]) it is difficult to generalize these findings to other contexts or populations of young adults. Regarding the other two previous longitudinal studies [34,44], they were conducted with samples of adults who exhibited different drinking patterns to those shown by young people [7,9].

The COVID-19 pandemic has been associated with increments in internalizing (depressive and anxiety) symptoms [49,50,51]. The self-medication theory has been proposed as a mechanism to explain the comorbidity between depression/anxiety and substance use [52]. This theory posits that people may use alcohol to improve mood and alleviate emotional distress (i.e., to cope with negative affect or emotions). This, in turn, promotes, via negative reinforcement and as supported by preclinical studies (see [53]), increased alcohol use. During the pandemic, those with more symptoms of depression [24,35,37,38,54] and anxiety [24,35,38,55,56] reported greater increases in alcohol consumption. Thus, anxiety and depression are two risk factors that may explain observed changes in alcohol use during the pandemic [20,21]. However, it is important to note that all these results on post-COVID-19 anxiety, depression and alcohol consumption come from retrospective cross-sectional studies.

The present study aimed to longitudinally assess (a) changes in alcohol use before and after COVID-19 outbreak in a community sample of Spanish young adults, and (b) the effect of sociodemographic variables (i.e., age, gender and college status) and the prospective impact of mental health status (i.e., anxiety and depression) before the COVID-19 outbreak on the shifts in alcohol use after the COVID-19 outbreak. This information could help reduce the impact of the pandemic and inform interventions in this and future public health crises. The aforementioned mixed results, regarding the impact of COVID-19 crisis on mental health and alcohol use behaviors, prompted us to follow an exploratory approach in this study, and no tentative hypothesis was formulated.

## 2. Materials and Methods

### 2.1. Participants and Procedure

The data for this longitudinal study were collected through a targeted sampling procedure [57] as part of a more extensive ongoing longitudinal study with a community sample of 360 young adults aged 18–25 years [58]. Between September to December 2019, participants were approached in a variety of community settings of the city of Huelva (Spain). To participate in the study, participants had to report alcohol use on two or more occasions during the past month. In this study, we used data from participants who responded to the first follow up, carried out two months after the baseline assessment, between November 2019 and February 2020 (i.e., pre-COVID-19 outbreak data), and the second follow-up, carried out one year after the COVID-19 outbreak during March 2021 (i.e., post-COVID-19 outbreak data). It should be noted that, in Spain, the initial country-wide lockdown began on 14 March 2020.

Similar to the baseline assessment, the first follow-up consisted of a self-administered paper-and-pencil questionnaire which was completed in a room of the University of Huelva. A mixed method procedure was employed [59] to contact participants for the first follow-up. Seven days before the exact date that they were supposed to complete the first follow-up questionnaire, participants received a pre-notification via instant messaging on their phone (i.e., WhatsApp message) and were informed that in 2–3 days they would receive a telephone call to request their participation and to schedule an appointment. In this call participants were also informed that they would be compensated with a 15-euro Amazon voucher. Those who did not respond were contacted two more times (via instant messaging and a telephone call). Eighteen participants refused to complete the first follow-up questionnaire, and three did not respond to any contact. Following procedures of previous research [60], and as evidence of validity of the responses, we asked participants about the use of a fictitious drug (Nadropax). None of the participants reported its use at the first follow-up. Almost all (i.e., 339 out of 360) participants that answered the baseline completed and had valid answers for the first follow-up questionnaire.

The second follow-up assessment consisted of an on-line questionnaire. For this follow-up, the 360 participants that completed the baseline assessment were contacted via a telephone call three/four days before sending them the email with the personal link to the questionnaire. In this call, they were informed about their receipt of the email, the duration of the questionnaire completion (around ten minutes) and that, after completion of the questionnaire, they would be compensated with a 5-euro Amazon voucher. Once the email was sent, participants were informed via instant messaging. Among those who did not answer to the questionnaire, three reminders were sent within the following week; specifically, two days (via WhatsApp message), four days (via WhatsApp message) and seven days (via telephone call) after the email was sent. A total of 330 participants answered the second follow-up questionnaire. None of them reported the use of Nadropax. Furthermore, considering the on-line nature of the second follow-up, a second question was included to obtain evidence of validity of the responses. The validity question stated: We want to confirm that you have read all the questions in this survey. If you have read all the questions, please, of the following options, select the last, “totally agree”. Of the 330 participants, 13 did not select the correct option and, therefore, their answers were discarded. A total of 317 participants completed and gave valid responses to the second follow-up questionnaire.

The final sample of the present study consisted of participants who answered both the first and second follow-up questionnaires (*n* = 305). Before completing each follow-up questionnaire, the participants provided informed consent. The protocol for this study was approved by the Regional Committee for Bioethics Research of Andalusia (Regional Ministry of Health, Andalusia, Spain).

### 2.2. Measures

The questionnaire gathered information on:

Sociodemographic characteristics (first follow-up): Age, gender, country of birth, main source of income, living arrangements, and college status (studying at university or not).

Alcohol use (first and second follow-up). In both assessments, information on the frequency (i.e., number of days) of alcohol use during past two months was collected. A modified version of the Daily Drinking Questionnaire (DDQ; [61]) was included to gather information about drinking quantity. Participants were questioned about the number of glasses consumed of six types of alcoholic beverages (accompanied by their images), as established by the Spanish Observatory of Drugs and Addictions [8], during a typical week in the past two months. The number of drinks consumed were converted into Standard Drink Units (SDUs, equivalent to 10 g of pure alcohol in Spain, [62]).

Anxiety and depression (first follow-up. To assess anxiety and depression, two sub-scales from the Brief Symptom Inventory (BSI-18) were used. The BSI-18 is a self-report scale of psychological distress designed by Derogatis [63]. It presents 18 items grouped into three subscales or dimensions (with six items each) that assess: somatization (i.e., distress caused by the perception of bodily dysfunction), depression (i.e., symptoms of disaffection and dysphoric mood), and anxiety (i.e., symptoms of nervousness, tension, motor restlessness, apprehension and panic). Each item is a statement that test-takers have to respond based on their level of distress over the preceding seven days using a five-point Likert scale (from 0 = not at all, to 4 = extremely). However, for the purpose of this study, the time-frame considered was the last two months in order to match with the time frame of reported alcohol use by the participants. Items of each subscale were summed (range = 0–24 for each subscale), with higher scores indicating higher anxiety, depression or somatization. Internal consistency estimated using Cronbach’s alpha was α = 0.85 for the Depression subscale and α = 0.74 for the anxiety subscale.

### 2.3. COVID-19 Context of the Study

During data collection for the second follow-up (i.e., post-COVID-19 outbreak assessment), Spain was under a so-called “state of alarm” [64,65], which implied various restrictions. In the Autonomous Region of Andalusia (where data collection was conducted) the restrictions imposed during the two months prior to data collection (January 15 to March 15; time frame for measures employed in this study) were as follows. First, a night-time curfew was stablished between 10 or 11 p.m. and 6 a.m. Social gatherings, both indoors and outdoors, were limited to no more than four to six people, and restaurants, bars and cafes had to close at 8 to 10 p.m. Finally, nonessential mobility of people between all the provinces of Andalusia and to other autonomous regions was limited, and municipalities exceeding a cumulative incidence of 500 cases per 100,000 inhabitants during the last 14 days were ordered to lock down.

### 2.4. Analytical Strategy

A linear mixed-effects model with a random intercept was used to address the aims of this study. First, a linear mixed-effects model that only included time as a predictor was estimated to evaluate changes in alcohol use before and after the COVID-19 outbreak. Then, a linear mixed-effects model that included all the predictors was estimated to analyze the prospective effect of sociodemographic and mental health variables on changes in alcohol use during the COVID-19 outbreak. The best fitting level 1 error–covariance structure was established using the Akaike’s Information Criteria. Two models were tested, one for the changes in the drinking frequency, and the other for changes in the quantity of alcohol consumed. Predictor variables included in the models were time (pre-post-COVID-19 outbreak), gender, age, college status, depression symptoms, and anxiety symptoms. Continuous variables were centered around the mean to facilitate interpretation. Categorical variables were dummy coded. Reference categories (coded as 0) were: pre-COVID time, males, and noncollege participants. The interaction between time and each predictor and the main effect of each predictor were introduced as fixed effects in the models. Main effects were interpreted as the effect of the predictors at pre-COVID-19 time. Interaction effects indicate if the changes from pre- to post-COVID-19 outbreak were associated to any of the predictors. A pick-a-point approach was used to facilitate interpretation of interactions that involved continuous variables (i.e., anxiety and depression symptoms) using the 16th, 50th and 84th percentiles as probing points (representing low, moderate and high levels of anxiety/depression). All analyses were conducted using SPSS 23.0. The Alpha value was set at 0.05.

## 3. Results

Table 1 shows descriptive data of the main variables of interest. The sample consisted of participants aged between 18 and 26 years (mean age = 21.27, (*SD* = 2.21)). More than half of them were female. Almost all participants were born in Spain (*n* = 297, 97.4%) and 61.6% were studying at university at the time of completing the first questionnaire. The main sources of income were family allowance (53.1%) and employment (22.6%), and most participants lived with parents (75.7%) or roommates (19.3%).

**Figure 1:**
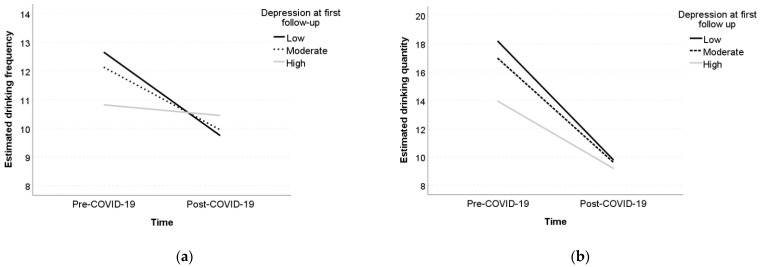
Simple slopes of the effect of time on drinking frequency (**a**) and drinking quantity (**b**) for selected levels of depression. Note: The depression levels correspond to the 16th, 50th, and 84th percentiles of the distribution of the depression scale describing relatively low, moderate and relatively high values of depression.

**Figure 2:**
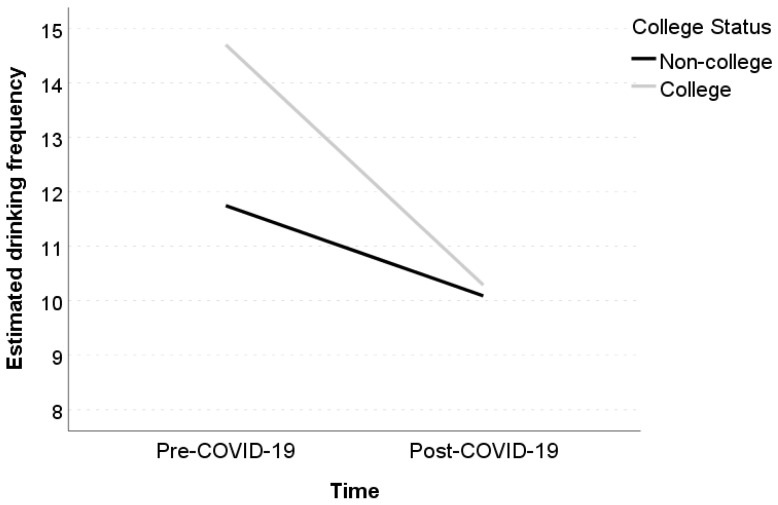
Simple slopes of the effect of time on drinking frequency as a function of college status.

The linear mixed-effects model with time as the only fixed predictor to estimate changes in drinking frequency revealed an average decrease of 3.16 days between pre- and post-COVID-19 outbreak (Table 2). The full linear mixed model with all predictors revealed significant main effects of gender (*b* = −2.21, *p* = 0.042) and college status (*b* = 2.95, *p* = 0.008) on drinking frequency. Specifically, we found that, at baseline, women drank on average 2.21 days less than men, and college students drank on average 2.95 days more than their noncollege peers. We also found a significant interaction between depression and time (*b* = 0.36, *p* = 0.038). As illustrated with the pick-a-point approach (Figure 1a), those with a relatively high depression level (*b* = −1.10, *p* = 0.306) did not show a decrease in drinking frequency, while those with a relatively low (*b* = −2.91, *p* = 0.014) or moderate depression level (*b* = −2.18, *p* = 0.039) exhibited significant declines in drinking frequency. A significant interaction between college status and time (*b* = −2.75, *p* = 0.016) was also found (see Figure 2). In particular, a significant decrease in drinking frequency was observed among college students (*b* = −4.41, *p* < 0.001), but not among their noncollege peers (*b* = −1.66, *p* = 0.107).

Regarding quantity of alcohol consumed by the participants in the previous two months, the linear mixed-effects model with time as the only fixed predictor to estimate changes in quantity of alcohol consumption revealed an average decrease of 6.44 SDUs between pre- and post-COVID-19 outbreak (Table 3). The linear mixed-effects model that included all predictors revealed a statistically significant main effect of anxiety (*b* = 0.67, *p* = 0.023). Those with higher levels of anxiety drank more alcohol before the COVID outbreak. However, a nonsignificant interactive effect between anxiety and time (*b* = −0.34, *p* = 0.23) was detected. A significant main effect of depression (*b* = −0.60, *p* = 0.012) and an interaction between depression and time (*b* = 0.52, *p* = 0.023) were found. As can be seen in Figure 1b, decreases in quantity of alcohol use were less pronounced among those with a relatively high level of depression (*b* = −4.71, *p* = 0.003) and more pronounced for those with relatively low (*b* = −8.32, *p* < 0.001) or moderate (*b* = −7.29, *p* < 0.001) level of depression.

## 4. Discussion

To our knowledge, this is the first study to longitudinally evaluate changes in alcohol consumption during the COVID-19 pandemic in a community sample of young adults while evaluating the predictive value of sociodemographic variables and mental health status on such changes. Our results show that the frequency and quantity of alcohol consumption decreased from pre to post-COVID-19 outbreak. A significant decrease in drinking frequency was observed among college students, but not in their noncollege peers. Although we found no effect of pre-COVID-19 anxiety on changes in alcohol use, those with more depressive symptoms at pre-COVID assessment were more resistant to decreasing their drinking quantity and frequency of alcohol use following the COVID-19 outbreak.

Consistent with other studies conducted with young people and adults [25,27,31,35], our results showed that both drinking quantity and frequency significantly decreased during the pandemic. Alcohol availability (i.e., the number of off-premise outlets such as supermarkets, and on-premise outlets such as bars, which sell alcoholic beverages in an area), was identified as an environmental factor associated with greater alcohol consumption [66,67,68], particularly among young people [66,69]. Thus, the overall reduction in drinking quantity and frequency could be due to the closure of on-premise alcohol outlets (i.e., bars, pubs and clubs), a measure applied to slow down the spread of the virus, which led to a decreased level of alcohol availability and reduced opportunities for drinking outside of the home [70].

Our results also showed that, as expected [71,72], men exhibited a higher frequency of drinking than women before the COVID-19 outbreak. However, like other studies among young people [27,29,30], we found no effect of gender on changes in drinking habits after the COVID-19 outbreak. Moreover, it was found that before the COVID-19 pandemic, college students consumed alcohol more frequently than noncollege students. However, interestingly, their drinking frequency dropped to the level of noncollege students during the COVID-19 pandemic. There is evidence that college students drink more and more frequently and exhibit more binge drinking episodes than their noncollege peers [3,6,73]. In addition, and consistent with our findings, some recent studies [25,27] have reported a decrease in alcohol consumption among college students during the pandemic. It should be noted that college attendance has been associated to greater alcohol consumption mainly due to contextual characteristics [74]. During college years, many social activities occur in contexts related to alcohol consumption [74] and the social interactions among college students seem to be facilitated by alcohol consumption [75,76]. Besides, it has been shown that before the pandemic, college student drinking primarily occurred in social contexts, such as in groups of friends [77]. During the pandemic, the suspension of college classes, campus closures and the transition to remote learning, were measures adopted by most countries to slow down the spread of the virus. In fact, during data collection, most of the Andalusian colleges had suspended face-to-face classes. These measures could have limited the opportunities for college students to drink, mainly due to a deprivation of the college context and the consequent reduction of associated social contact and alcohol-related activities.

During the pandemic, increases in alcohol consumption have been hypothesized as mechanisms for coping with the anxiety, depression and social isolation induced by the measures applied to slow down the spread of the virus [70]. Unlike previous studies [24], we found no relationship between depression and increases in alcohol consumption during the pandemic. However, our results highlight the role of depression as a risk factor for alcohol consumption during the pandemic, since despite not being associated with an increase in alcohol consumption, those with higher levels of depression before the pandemic were less likely to reduce their alcohol consumption. These results are consistent with the premises of the self-medication theory [52] and are in line with findings concerning other mental health variables such as stress [31].

Previous studies [24,35,56] showed that anxiety increased alcohol use during the pandemic, with the latter being considered a potentially anxiety-inducing scenario, particularly for young people [78]. However, our results do not support these previous research findings. This may be due to the impact of other variables not measured here, such as family structure (e.g., having children living at home) [35,79], or social support [78,79], which could have moderated the impact of the pandemic on mental health and thus its relationship with alcohol use. Another possible explanation could be based on the type of anxiety measured here. Although more research is needed, it is possible that among young people, anxiety is more significant in social interactions, which were drastically reduced during the pandemic, possibly lessening the impact of anxiety on drinking behavior. In fact, it has been pointed out that social anxiety, but not clinical anxiety, is related to alcohol use among college students [80]. Another possible explanation for the lack of association between pre-COVID-19 anxiety level and changes in alcohol use refers to the timing of the measurement of these variables. In the present study, anxiety was measured before the pandemic outbreak. It is possible that pre-COVID-19 anxiety is, compared to anxiety levels during the first weeks of the pandemic (i.e., when lockdown and stay-at-home orders were in force), a lesser predictor of alcohol use during the pandemic. In this regard, a cohort study [23] showed that anxiety increased during the first weeks of the pandemic but returned to pre-COVID-19 levels a few months later, despite the persistence of restrictions and social distancing measures.

Some limitations of this study must be considered. First, although the study provides pre- and post- COVID-19 outbreak information, all data were self-reported, which can affect the validity of our results due to recall bias. Moreover, although the models predicting changes in drinking frequency and drinking quantity were statistically significant, the amount of variance explained was relatively low, limiting the scope of our results. Further, a nonprobabilistic sampling procedure was used, which limits the representativeness of our sample and the possibility of generalizing our results to other Spanish youths. Furthermore, data collection procedures included face-to-face (pre-COVID-19 outbreak data) and online data collection (post-COVID-19 outbreak data). These differences may affect their comparability and, therefore, the validity of our findings. To reduce the impact of measurement differences across the survey, however, we followed the recommendations of Dillman et al. [59]. For instance, we used the same question wording and visual format across modes. Finally, and as it has been pointed out, post-COVID-19 outbreak depression and anxiety measures were not included, limiting the possibility of analyzing whether changes in mental health status (and not only the prospective effect of baseline levels of anxiety and depression) were related to changes in alcohol consumption.

In spite of these limitations, the present results provide valuable information that partially support the self-medication theory [52]. We found that although an overall pre-post COVID-19 decrease in alcohol use was found, those with higher levels of depression (but not anxiety) at the pre-COVID assessment were more resistant to decreasing their drinking during the pandemic. This information should help in designing interventions aimed at reducing harmful alcohol use and its impact during this, and future, public health crises. In light of our findings, mental health status should be considered when identifying high risk populations of young adults during health crises.

## Figures and Tables

**Table 1:** Sample characteristics.

	%/*M* ± *SD*	
**Gender (men)**	46.56	
**Age**	21.27 ± 2.21	
**Country of birth**		
Spain	97.38	
Other European country	1.31	
Other country (non-European)	1.31	
**College status (college student)**	61.64	
**Main source of income**		
Family allowance	53.11	
Employment	22.62	
Scholarship	20.99	
Unemployment insurance	0.66	
Other	2.62	
**Model of cohabitation**		
Parents	75.74	
Roommates	19.34	
Friends	2.62	
Alone	1.31	
Partner	0.99	
**Mental health**		
Depression	3.45 ± 4.34	
Anxiety	2.76 ± 3.50	
**Alcohol Use**	**Pre-COVID-19 outbreak**	**Post-COVID-19 outbreak**
Drinking days	12.37 ± 9.69	9.18 ± 9.26
Drinking quantity (typical week)	15.41 ± 13.29	9 ± 9.45

**Table 2:** Linear mixed models for drinking frequency outcome.

	Time-Only Model			Full Model		
	Estimate (*SE*)	*t*	*p*	Estimate (*SE*)	*t*	*p*
Intercept	**12.38 (0.55)**	**22.69**	**0.000**	**11.75 (1.00)**	**11.71**	**0.000**
Time	**−3.16 (0.56)**	**5.63**	**0.000**	−1.66 (1.03)	−1.61	0.107
Gender				**−2.21 (1.09)**	**−2.04**	**0.042**
College Status				**2.95 (1.11)**	**2.66**	**0.008**
Age				0.13 (0.25)	0.53	0.596
Depression				−0.26 (0.17)	−1.54	0.123
Anxiety				0.29 (0.21)	1.37	0.172
Time*Anxiety				−0.24 (0.21)	−1.13	0.261
Time*Depression				**0.36 (0.17)**	**2.08**	**0.038**
Time*Age				−0.42 (0.25)	−1.69	0.093
Time*College Status				**−2.75 (1.14)**	**−2.42**	**0.016**
Time*Gender				0.35 (1.11)	0.32	0.751
	Estimate (*SE*)	Wald *z*	*p*	Estimate (*SE*)	Wald *z*	*p*
Level-1 errors						
Variance	47.70 (3.88)	12.29	0.000	45.62 (3.71)	12.29	0.000
Level-2 errors						
Intercept Variance	42.19 (5.71)	7.38	0.000	41.61 (5.56)	7.48	0.000
Akaike Information Criteria	4364.09			4363.08		
Total *R*^2^	0.030			0.056		

Reference categories (coded as 0) were: pre-COVID time, males, and noncollege participants. Significant results are indicated in bold font. Interaction terms are indicated with *.

**Table 3:** Linear mixed models for drinking quantity outcome.

	Time-Only Model			Full Model		
	Estimate (*SE*)	*t*	*p*	Estimate (*SE*)	*t*	*p*
Intercept	**15.48 (0.77)**	**20.21**	**0.000**	**16.07 (1.41)**	**11.42**	**0.000**
Time	**−6.44 (0.72)**	**8.89**	**0.000**	**−6.54 (1.33)**	**−4.91**	**0.000**
Gender				−1.60 (1.53)	−1.05	0.295
College Status				0.43 (1.56)	0.27	0.784
Age				−0.31 (0.35)	−0.89	0.375
Depression				**−0.60 (0.24)**	**−2.53**	**0.012**
Anxiety				**0.67 (0.29)**	**2.29**	**0.023**
Time*Anxiety				−0.34 (0.28)	−1.20	0.231
Time*Depression				**0.52 (0.23)**	**2.28**	**0.023**
Time*Age				−0.24 (0.33)	−0.74	0.457
Time*College Status				0.95 (1.48)	0.64	0.522
Time*Gender				−0.89 (1.45)	−0.61	0.540
	Estimate (*SE*)	Wald *z*	*p*	Estimate (*SE*)	Wald *z*	*p*
Level-1 errors						
Pre-COVID-19 Variance	121.88 (11.91)	10.23	0.000	119.68 (11.64)	10.28	0.000
Post-COVID-19 Variance	35.29 (7.13)	4.95	0.000	34.05 (6.88)	4.95	0.000
Level-2 errors						
Intercept Variance	54.34 (7.88)	6.89	0.000	51.45 (7.57)	6.80	0.000
Akaike Information Criteria	4565.53			4564.69		
Total *R*^2^	0.076			0.105		

Reference categories (coded as 0) were: pre-COVID time, males, and noncollege participants. Significant results are indicated in bold font. Interaction terms are indicated with *.

## Data Availability

Materials and data used in this manuscript are available by emailing the corresponding author.
